# Bandgap adjustment assisted preparation of >18% Cs_*y*_FA_1−*y*_PbI_*x*_Br_3−*x*_-based perovskite solar cells using a hybrid spraying process†

**DOI:** 10.1039/d1ra02666f

**Published:** 2021-05-13

**Authors:** Shengquan Fu, Yueyue Xiao, Xinxin Yu, Tianxing Xiang, Fei Long, Junyan Xiao, Zhiliang Ku, Jie Zhong, Wei Li, Fuzhi Huang, Yong Peng, Yibing Cheng

**Affiliations:** State Key Lab of Advanced Technologies for Materials Synthesis and Processing, Wuhan University of Technology Wuhan 430070 P. R. China yongpeng@whut.edu.cn; Wuhan University of Technology Advanced Engineering Technology Research Institute of Zhongshan City Zhongshan 528400 P. R. China; Guangxi Key Lab Opt & Elect Mat & Devices, Coll Mat Sci & Engn, Guilin University Technology Guilin 541004 P. R. China

## Abstract

The preparation of Cs_*y*_FA_1−*y*_PbI_*x*_Br_3−*x*_-based perovskite by ultrasonic spraying has valuable application in the preparation of tandem solar cells on textured substrates due to its excellent stability and the advantages of large-area uniform preparation from the spraying technology. However, the bandgap of perovskite prepared by spraying method is difficult to adjust, and perovskites with a wide bandgap have the issue of phase instability. Here, we improved the crystallinity of the perovskite by simply controlling the post-annealing temperature. The results show that perovskite film prepared by hybrid spray method has the best crystallinity and device performance at a post-annealing temperature of 170 °C. On this basis, the bandgap of perovskite was changed from 1.53 eV to 1.76 eV by controlling the ratio of the organic halogen precursor solution. When the bandgap is 1.57 eV, a perovskite solar cell with an efficiency of 18.31% is obtained.

## Abstract 2

High-efficiency perovskite solar cells with good grain morphology and adjustable band gap were prepared by ultrasonic spray.

## Graphical abstract

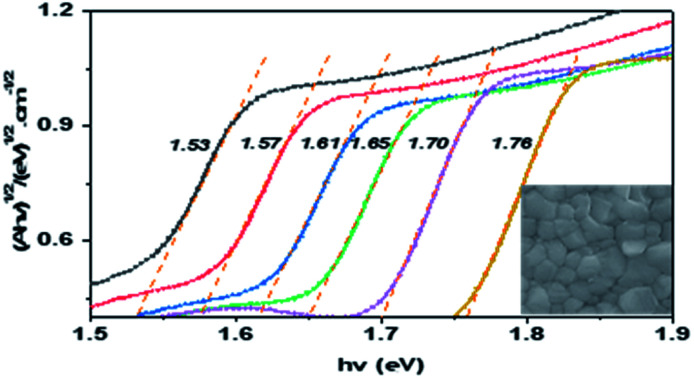

## Introduction

Perovskite/Si tandem solar cells have been attracting worldwide attention in recent years.^1–4^ One of the most important absorber candidates for top sub-cells is the Cs_*y*_FA_1−*y*_PbI_*x*_Br_3−*x*_-based perovskite because of its balanced properties in optical-electric performance and thermal stability.^5–10^ Spray technologies^11–13^ are a type of thin-film technology desirable for industrial purposes. Since the spray technologies were firstly introduced for perovskite by Lidzey^14^*et al.* in 2014, they have undergone quick development. In 2016, Heo *et al.* prepared MAPbI_3−*x*_Cl_*x*_ mixed halide perovskite solar cells with a PCE of 18.3% (ref. 15) by redissolution and grain growth. In 2019, Qi *et al.* fabricated FAPb(I_0.85_Br_0.15_)_3_ perovskite cells with great stability and a PCE of 16.2% *via* a two-step method of spraying and chemical vapor deposition.^16^ Other specific advances in perovskite solar cell preparation by spraying can be seen in Table S1.† Last year, we reported a hybrid spraying technology by which a Cs-FA-based PSC with a PCE of 18.2% was obtained.^17^

Intrinsically, this hybrid spray technology is a sequential deposition method. It first evaporates the metal halide inorganic layer and then sprays the organic halogen layer. Unlike the two-step solution method that can precisely control the elements in perovskite by controlling the concentration of the precursor solution and the rotation speed of spin coating, it is difficult to control the elements in perovskite films with high accuracy in the hybrid spray process. However, bandgap change, relating to the halide content in metal halide perovskite, is necessary for tandem cell applications.^18,19^ Most recently, it was found that wide-bandgap perovskite suffers from Br-rich phase-separation-related stability issues. Barker *et al.* studied the phase segregation behavior of MAPb(I_1−*X*_Br_*X*_)_3_ and found that ion segregation takes place *via* halide defects.^20^ Kim *et al.* reported a strategy to inhibit phase separation by improving the crystallinity of perovskite.^21^

In this research, through an evaporation/spray deposition method, high-efficiency Cs-FA perovskite thin film cells were prepared. CsBr/PbI_2_ composite film was first vacuum deposited on the substrate, then the mixed solution of FABr/FAI was sprayed ultrasonically. We first studied the effect of annealing temperature on the film and confirmed the formation of a high-quality perovskite film with good transformation and smooth surface by X-ray diffraction, scanning electron microscopy (SEM), photoluminescence (PL), space-charge-limited current (SCLC) and other characterization methods. In order to expand the application of perovskite cells, for example, achieve bandgap matching, they can be combined in series with the bottom cells of different bandgaps to form tandem cells. We changed the halogen element ratio in the perovskite grains by changing the Br/I ratio in the spray solution to achieve precise adjustment of the 1.53–1.76 eV bandgap of the perovskite thin film. Finally, the PCE of the perovskite cell with 1.57 eV bandgap under 0.15 cm^2^ area was achieved at 18.31%.

## Experimental

### Materials

Lead iodide, cesium bromide, formamidinium iodide, and formamidinium bromide were purchased from Xi'an *p*-OLED Corp; SnCl_2_·2H_2_O was from Aladdin. Spiro-MeOTAD was purchased from Shenzhen Feiming Science and Technology Co., Ltd. All other solvents and reagents were obtained from Sigma-Aldrich or Alfa Aesar and used as received.

### Device assembly

Femtosecond laser etching on FTO glass. The FTO glass was cleaned with deionized water and anhydrous ethanol in a cleaning solution, and then cleaned using the ultrasonic cleaning machine for 15 min. A 0.012 M SnCl_2_ solution was prepared, including 400 mL deionized water, 5 g urea, 5 mL 37 wt% HCl and 100 uL mercaptoacetic acid. The cleaned FTO glass was placed into a stannous chloride solution and kept in a 90 °C oven for 3 h. The treated FTO glass was cleaned with deionized water and blow-dried. A 30 nm CsBr film was deposited at a deposition speed of 0.3 Å s^−1^, and 300 nm PbI_2_ film was deposited at a speed of about 3 Å s^−1^ on glass/FTO/SnO_2_ substrates. The composite film from vacuum deposition was placed on a hot plate preheated to 130 °C, and the distance was set to 85 mm between the nozzle and the CsBr/PbI_2_ film. Different proportions of 20 mg FABr/FAI dissolved in 1 mL IPA solution were sprayed at 0.3 mL min^−1^ flow rate, with the carrier gas (pure nitrogen) velocity set to 1.2 L min^−1^. The perovskite wet layer was placed on the hot plate and annealed for 1 h. A hole-transporting layer (HTL) composed of 36.5 mg Spiro-MeOTAD was prepared with 14.5 μL FK209 solution, 9 μL Li-TFSI salt solution and 15 μL 4-*tert*-butylpridine dissolved in 1 mL ethyl acetate. A HTL layer was spin coated on the perovskite film. Finally, 100 nm Au was deposited on the HTL as the positive electrode. IPA mixed solutions with different FABr and FAI molar ratios were prepared to adjust the bandgap. The molar ratios of FABr and FAI in the mixed solution were divided into six proportions: 0 : 5 (*M* = 1), 1 : 4 (*M* = 0.8), 2 : 3 (*M* = 0.6), 3 : 2 (*M* = 0.4), 4 : 1 (*M* = 0.2) and 5 : 0 (*M* = 0).

### Device characterization

The X-ray diffraction (XRD) patterns of the perovskite films were measured using an X-ray diffractometer (Bruker D8 Advance) with Cu Kα radiation (*λ* = 1.54 Å). The morphologies of perovskite and other films were observed using field-emission scanning electron microscopy (FE-SEM, Zeiss Ultra Plus). The UV-vis absorption curves of perovskite films were recorded (lambda 750S, PerkinElmer). The electrochemical impedance spectroscopy (EIS) data were measured using EC-Lab (SP300) under light, with the measurement frequency range of 0.5 Hz to 2 MHz. Photoluminescence (PL) spectra were obtained by a fluorescence spectrometer (QE65 Pro, Ocean Optics). Under 100 mW cm^−2^ illumination (AM 1.5G) condition, the current density–voltage (*J*–*V*) curves of solar cells were estimated with a solar simulator (Oriel 94023A, 300 W) and a source meter (Keithley 2400), with forward scan from −0.1 to 1.2 V and then the reverse scan, both with a rate of 10 mV s^−1^. The dark *J*–*V* characteristics were investigated in an opaque black box; the reverse range was −0.75 to 1.0 V and the scan rate was 0.1 V s^−1^. The space-charge-limited current (SCLC) was also tested in an opaque black box, with −0.1 to 8.0 V range. The PSCs' defined active area was 0.15 cm^2^, measured in a black aperture without encapsulation. The external quantum efficiency (EQE) spectra were tested between 280–850 nm under dark condition by a commercial EQE system (Newport). The long-term PCEs were tested in ambient air under 25–35% relative humidity (RH) without light stimulation.

## Results and discussion

**Fig. 1:**
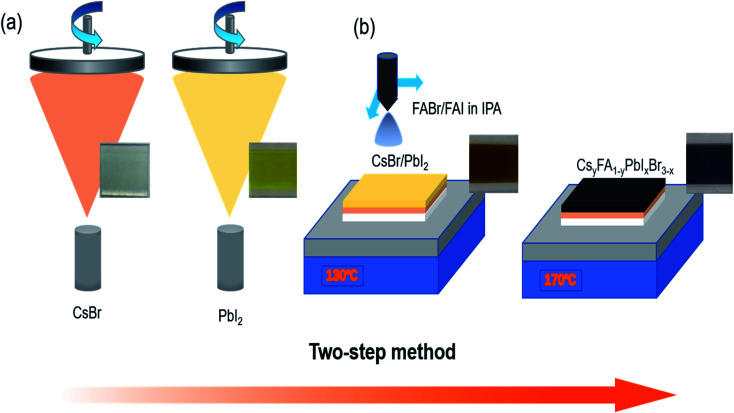
Schematic illustration of the two-step preparation of the Cs_*y*_FA_1−*y*_PbI_*x*_Br_3−*x*_ perovskite and photographs at each step: physical vapor deposition of CsBr and PbI_2_ and ultrasonic spraying of the FAI and FABr mixed solution.

**Fig. 2:**
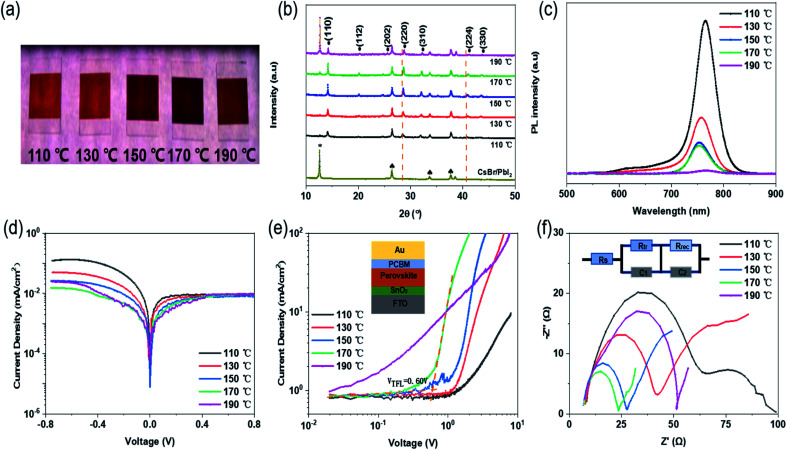
(a) Photographs of perovskite films post-annealed at 110 °C, 130 °C, 150 °C, 170 °C and 190 °C. (b) XRD spectrum for perovskite films at different post-annealing temperatures and for the CsBr/PbI_2_ composite film: solid round (•), plum blossom (*) and spade (♠) represent perovskite phase, PbI_2_ and FTO, respectively. (c) Steady-state PL spectra of perovskite films prepared at various post-annealing temperatures. (d) Semilog *J*–*V* curve and (e) double-log *J*–*V* curve of electron-only structure in the dark. (f) Nyquist plot of EIS spectra of the device.

Fig. 1 illustrates the fabrication of perovskite film. First, CsBr and PbI_2_ were deposited successively on FTO/glass substrate by vacuum evaporation. Then, the composite film was placed on a hot plate at 130 °C, and the mixed solution of FABr and FAI dissolved in IPA was ultrasonically sprayed on it. The yellow composite film became a light-brown wet film. Finally, the wet films obtained after spraying were placed on a hot plate for post-annealing treatment; the annealing time for all was 1 h. During the heat treatment process, the film gradually turned red-brown, as shown in Fig. 1. More details can be found in the experimental section. The post-annealing temperature has an important influence on the crystallization kinetics of perovskite. Barrows *et al.* proved that the post-annealing temperature determines the grain morphology and microstructure of the film in ultrasonic spray. Low annealing temperature is not conducive to the crystallization of perovskite crystals, and a series of pores are produced in the resulting perovskite grains. Better perovskite grains are obtained at the appropriate annealing temperature.^22^ In this way, in the process of solution reaction, the solvent can be timely evaporated cleanly to form a high-quality film.^23^ Therefore, five different post-annealing temperatures (110 °C, 130 °C, 150 °C, 170 °C and 190 °C) were set here to observe the effect of different annealing temperatures on the morphology and crystallinity of perovskite films. The results show that different colours of perovskite phase can be obtained at different post-annealing temperatures. Within 110–170 °C, with the increase of temperature, the perovskite surface shows obvious colour deepening, as shown in Fig. 2(a). When the temperature exceeds 170 °C and reaches 190 °C, the colour of perovskite changes from dark to light, revealing significant thermal decomposition of perovskites.

Fig. 2(b) reveals the XRD patterns of perovskite prepared at 110–190 °C and the CsBr/PbI_2_ composite film. All the samples, except the CsBr/PbI_2_ film, present similar diffraction patterns, which indicates that the perovskite films prepared at different temperatures had the same crystalline structure. In addition to FTO and PbI_2_ peaks, the XRD diffraction peaks correspond to (110), (112), (202), (220), (310), (224) and (330) planes of the perovskite structure. With the elevation of temperature, when the post-annealing temperature is below 170 °C, the intensity of all diffraction peaks of the perovskite film gradually increases. The perovskite annealed at 190 °C shows smaller perovskite peaks and particularly more peaks of PbI_2_.^24,25^ It is proved that a certain high annealing temperature is conducive to crystallization, and a temperature above 190 °C will lead to perovskite decomposition. With the rise of annealing temperature, the (220) and (224) planes show a slight shift to higher angles, which may be attributed to the presence of residual stress in perovskite grains.^26–28^

**Fig. 3:**
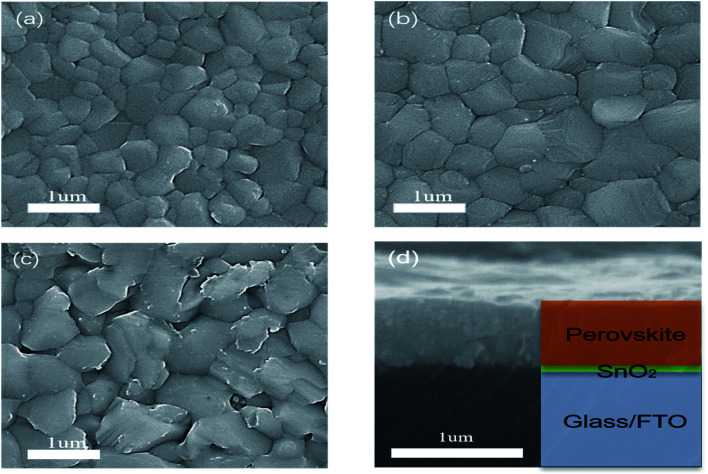
SEM images of surface morphology of perovskite annealed at (a) 150 °C, (b) 170 °C and (c) 190 °C. (d) The cross-section of perovskite film on SnO_2_/FTO/glass.

SEM was carried out to further prove the effect of post-annealing temperature on the crystallinity of the perovskite film. As can be seen from Fig. 3 and S1,† with the enhancement of annealing temperature, the grain size increases significantly, and the grain diameter grows from about 200 nm to more than 1 um. When the annealing temperature is 110 °C and 130 °C, the film morphology shows the individual large grain surrounded by a large number of small grains, which may be due to the slow evaporation of solvent caused by the lower temperature in the heating process. It is proved that low temperature does not benefit solvent evaporation in forming the film. With the increase of temperature, the grain size generated at 130 °C is generally close to 400 nm, and there are fewer large-sized grains. At 170 °C, the grain size has grown to about 600–800 nm. The grain boundary is obvious, and the film is compact and uniform without pinholes, reaching a relatively good state. From 110 to 170 °C, the surface morphology and roughness of the perovskite film are also gradually improved. However, when the temperature reaches 190 °C, the excessive thermal energy promotes large grains and also leads to the decomposition of perovskite. Accompanied by the formation of more PbI_2_, a number of holes appear on the surface of the perovskite film, affecting the performance of the solar cells. Meanwhile, the perovskite film prepared at 170 °C has none of the coffee rings or holes commonly seen in ultrasonic spray.^29^ From the cross-sectional SEM picture shown in Fig. 3(d), it is closely fitted, and there is no gap between the perovskite film and FTO/SnO_2_ film. These all help to improve the performance of the perovskite solar cells. Steady-state PL measurement was conducted to further confirm the crystal quality of the perovskite films fabricated at various post-annealing temperatures. As shown in Fig. 2(c), the emission peaks at approximately 770 nm correspond to the bandgap of Cs_*y*_FA_1−*y*_PbI_*x*_Br_3−*x*_-based perovskite. In addition, it can be seen from the PL peak intensity that the photoluminescence intensity decreases with the increase of post-annealing temperature.

Table 1 shows the photovoltaic performance of solar cells prepared at different annealing temperatures. All cell parameters depend on the annealing temperature of the substrate. It is obvious that in the 110 to 170 °C range, accompanied by an increase in annealing temperature, the open-circuit voltage (*V*_oc_), the current density (*J*_sc_) and the fill factor (FF) have different amplitudes of enhancement. Among them, the current density shows the highest increase, improving from 1.84 mA cm^−2^ at 110 °C to 21.56 mA cm^−2^ at 170 °C. The most important reason is that the residual solvent in the wet film evaporates in time under appropriate temperature, and the perovskite film grows a dense morphology and suitable grains, which promotes electron–hole transmission. The XRD patterns show that when annealing at 170 °C, a small amount of PbI_2_ is retained, reducing the charge separation from defect states and the surface recombination, increasing carrier life and forming a passivation modification effect on the film.^30,31^ At this point, the *V*_oc_ is 1071 mV, the *J*_sc_ is 21.56 mA cm^−2^, and the efficiency reaches the maximum of 17.36%. However, when the temperature reaches 190 °C, excess heat destroys the grain composition, resulting in a large number of PbI_2_ precipitation in the bulk and interfaces. The grain boundaries, the defects situated in bulk, and interfaces usually facilitate ion migration.^30,32,33^ They act as charge carrier recombination sites, leading to degradation of perovskite film and efficiency loss in the device.^34^

**Table 1:** Photoelectric parameters of devices prepared at different annealing temperatures

*T* (°C)	Scan direction	*V*_OC_ (mV)	*J*_SC_ (mA cm^−2^)	FF (%)	PCE (%)
110 °C	Reverse	979	1.84	47	0.85
	Forward	960	1.6	42	0.64
130 °C	Reverse	1005	6.88	48	3.29
	Forward	980	5.4	42	2.23
150 °C	Reverse	1047	18.78	60	11.81
	Forward	1006	18.72	49	9.26
170 °C	Reverse	1071	21.56	75	17.36
	Forward	1018	21.55	65	14.28
190 °C	Reverse	862	16.65	53	7.66
	Forward	780	15.12	21	2.5

From the dark *J*–*V* characteristics of the cells prepared at different annealing temperatures in Fig. 2(d), it can be seen that the dark currents of the cells decrease gradually with the increase of temperature. When the temperature is 170 °C, the leakage current is the lowest. On the contrary, the current at 190 °C shows an unusual increase, due to the high temperature leading to the decomposition of perovskite and the large leakage current.

In order to explain more directly the effect of annealing temperature on perovskite grain growth, electron-only structures with FTO/SnO_2_/Perovskite/PCBM/Au were prepared. In Fig. 2(e), the trap-filled voltage (*V*_TFL_) can be found between the ohmic region and the TFL region through the space-charge-limited current (SCLC) method. It can be clearly observed that with the increase of temperature, the *V*_TFL_ values of the devices decrease to the minimum value (*V*_TFL_ = 0.60 V) at 170 °C. The trap state density of the cell is also the lowest, which indicates that the larger grain size and better crystal surface morphology are more conducive to the improvement of interface contact between the perovskite layer and the charge transport layer. Fig. 2(f) shows the impedance characteristics (and equivalent circuit) of the cells analyzed by EIS, where the measurement frequency range was 0.5 Hz to 2 MHz. The semicircles in the Nyquist plots represent the impedance of the carrier transport between the electron transport layer/perovskite layer/hole transport layer. The larger the impedance, the slower the carrier transmission.^35,36^ The experimental results show that the charge transport resistance (*R*_tr_) decreases significantly with the increase of annealing temperature, reaching the minimum at 170 °C and increasing again at 190 °C. The main reason is that the decomposition of perovskite film destroys the carrier transport. The trend of carrier recombination resistance (*R*_rec_) is similar. It is proved again that the perovskite film made at 170 °C helps in charge collection and transport, and effectively reduces the recombination of charge carriers, thus improving the efficiency of the solar cells.

**Fig. 4:**
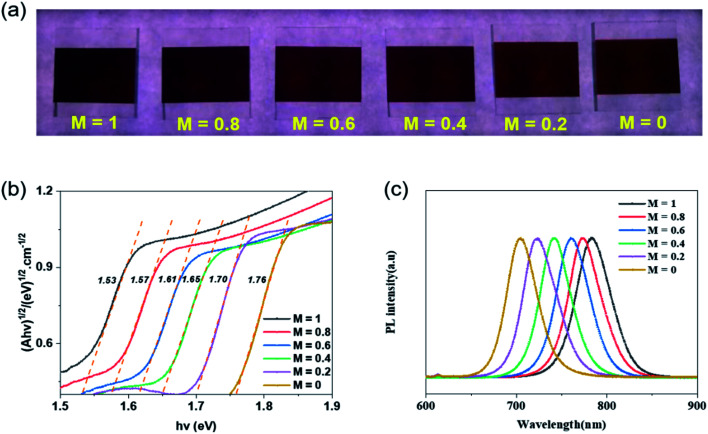
(a) The perovskite films prepared *via* CsBr/PbI_2_ composite film sprayed with IPA solutions of different Br/I ratios (0 : 5, 1 : 4, 2 : 3, 3 : 2, 4 : 1, 5 : 0 from left to right). (b) Tauc plot of Cs_*y*_FA_1−*y*_PbI_*x*_Br_3−*x*_ thin films with different Br contents. (c) Steady-state PL spectra of perovskite films prepared with various Br contents.

The above characterizations indicate that 170 °C post-treatment is conducive to the crystallization of perovskite film prepared by ultrasonic spray and is beneficial to obtain a good device performance. On this basis, we continued to adjust the bandgap of perovskite thin films, in order to obtain a wide-bandgap perovskite film and high-efficiency device. The halogen content plays a direct role in adjusting the bandgap of metal halide perovskite. Based on 170 °C post-treatment temperature, there was no gaseous phase substance, for example FABr or FAI, released during the reaction process. Therefore, the proportion of Br and I in the generated perovskite was basically the same as the content added. The perovskite films with different bandgaps were generated. It is apparent from the photographs (Fig. 4(a)) that the perovskite films prepared with different bandgaps are shown in different colours under the light. From left to right, the light changes from dark-brown to light-brown, proving that the bandgaps of perovskite affect the degree of light absorption by the film. The corresponding perovskite film absorptions, in Fig. S2(a),† were tested using a UV-vis spectrophotometer. With the increase of Br content, the absorption limit of perovskite film significantly shifted to blue. When *M* = 1, the absorption edge of films is located at 810 nm; when *M* = 0.6, the edge of perovskite films moves to 742 nm, and when *M* = 0, it reaches 704 nm. For semiconductor materials, the bandgap of the material can be calculated according to the absorption curve of the material. The specific calculation formula is in eqn (S1) and (S2).†

Consequently, the corresponding bandgaps were measured, as shown in Fig. 4(b). On the basis of CsBr/PbI_2_ composite film, when *M* = 1, the bandgap of Cs_*y*_FA_1−*y*_PbI_*x*_Br_3−*x*_ is 1.53 eV. Then with the sequential change of Br/I, the bandgap raises each time. It peaks at 1.76 eV. As can be seen from Fig. 4(c), similar significant PL peaks can be observed in the range of 650–850 nm. With the increase of Br content in the perovskite film, the PL peaks show blue shift, which is the same as the change of *E*_g_.

**Fig. 5:**
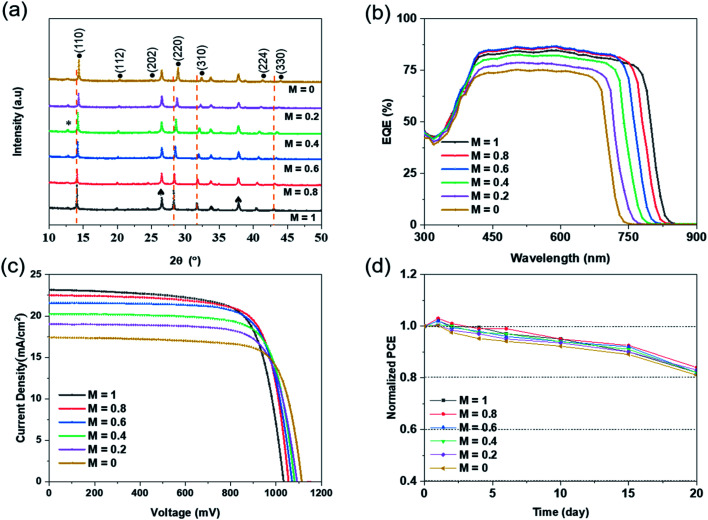
(a) XRD spectrum for perovskite films at different Br contents and for the CsBr/PbI_2_ composite film; solid round (•), plum blossom (⁎) and spade (♠) represent perovskite phase, PbI_2_ and FTO, respectively. (b) EQE and (c) the *J*–*V* curves of the device. (d) Long-term PCE performance for the device in ambient air under RH of 25–35% for 20 days.

Through the XRD pattern of perovskite in Fig. 5(a), it can be found that adjusting the solution content ratio will not affect the generation of perovskite. All the composite films reacted completely with the IPA mixed solution to produce perovskite films, without significant residue. Meanwhile, with the increase of Br ion content, all main perovskite diffraction peaks almost correspondingly move towards a larger angle. By Bragg's law, in eqn (S3),† the radius of Br ions is smaller than that of I ions. With the augment of Br content in the IPA reaction solution, the *X* position in the perovskite lattice is more occupied by Br element, which leads to the decrease of *d* value and the increase of *θ* angle. Therefore, the main peaks of perovskite shifted right.

Table 2 shows the performance parameters of perovskite solar cells with different bandgaps prepared by different Br/I ratios, and Fig. 5(c) presents the *J*–*V* curve of the corresponding perovskite cells. As the bandgap becomes wider, the voltage of the prepared cell devices increases gradually, and the current density decreases continuously. In theory, the *V*_oc_ of the cells should be the same as the bandgap of the perovskite film. It can be seen that the voltage of the solar cell is 1022 mV at the narrowest bandgap of 1.53 eV, and 1130 mV at the widest bandgap of 1.76 eV. As for the dark *J*–*V* characteristics in Fig. S2(b),† the dark currents of perovskite cells with different Br/I ratios are similar, except that the current leakage is greater when *M* = 0.2 or 0. Although the voltage increases gradually, there is still a big deficiency between the actual voltage and the theoretical *V*_oc_. It is highly likely that defects in the preparation of the cell, as well as mismatches between the perovskite and other functional films, result in a loss of voltage.

**Table 2:** Photoelectric parameters of perovskite solar cells prepared by different Br/I ratios

FABr : FAI		*E*_g_ (eV)	Scan direction	*V*_OC_ (mV)	*J*_SC_ (mA cm^−2^)	FF (%)	PCE (%)
0 : 5	*M* = 1	1.53	Reverse	1022	23.17	74	17.52
			Forward	996	22.12	66	14.54
1 : 4	*M* = 0.8	1.57	Reverse	1056	22.52	77	18.31
			Forward	1022	22.34	63	14.38
2 : 3	*M* = 0.6	1.61	Reverse	1071	21.56	75	17.36
			Forward	1018	21.55	65	14.28
3 : 2	*M* = 0.4	1.65	Reverse	1097	20.23	76	16.8
			Forward	1046	20.11	62	13.04
4 : 1	*M* = 0.2	1.7	Reverse	1102	19.01	75	15.79
			Forward	1057	18.99	65	13.05
5 : 0	*M* = 0	1.76	Reverse	1130	17.41	76	15.03
			Forward	1091	17.28	64	12.07

External quantum efficiency (EQE) is one of the main performance indicators of photoelectric devices. It is expressed as the ratio of the number of electrons collected to the number of electrons incident. Due to the nature of photoelectric semiconductor materials, the energy of the incident photoelectron must exceed the bandgap of the semiconductor. In this way, there is enough energy to excite the electrons in the valence band into the conduction band, generating electron–hole pairs, and thus forming current. Therefore, different gaps have different requirements for photoelectron energy, which correspond to different wavelengths of light. It can be clearly seen in Fig. 5(b) that with the broadening of the bandgap, the limit edge of light moves towards the narrow band. The wavelength has been reduced from a maximum of about 800 nm to 700 nm, which represents the increasing energy of photoelectrons and the improving minimum standard for generating photocurrent. There are differences in the matching of energy levels between perovskite film and other functional layers, due to the same functional layers resulting in different photoelectron absorptions. When the bandgaps are 1.53 eV, 1.57 eV, and 1.61 eV, the photoelectron absorption of the cell is high, above 80%. The generated currents are 23.17 mA cm^−2^, 22.52 mA cm^−2^ and 21.56 mA cm^−2^, respectively. The PCEs of the cells are all above 17%. When the bandgaps further widen, the photonic electron absorption of the cells gradually weakens, and the EQE value significantly decreases. The current density dropped from 20.23 mA cm^−2^ to 17.41 mA cm^−2^, and the efficiency of the cells dropped to about 15%. Finally, when *M* = 0.8, the bandgap is 1.57 eV, the voltage reaches 1056 mV, the current 22.52 mA cm^−2^, the FF is 0.77, and the perovskite cell obtains the highest efficiency of 18.31%.

In addition to performance, the stability of solar cells is also important. In the experiment, unencapsulated perovskite cells with different bandgaps were placed in air under RH of 25–35% for 20 days to measure the stability of the devices. In Fig. 5(d), it can be seen that the PCEs of all perovskites have been slightly improved within the first 1 to 2 days. The possible reason is the oxidation of the HTL (Spiro-MeOTAD), which improves the transmission of the holes and promotes electron–hole recombination in the cells. But as time goes on, the cell performance continues to decline. After 15 days, the PCE of the cells retains about 90% of the initial efficiency, and after 20 days, it drops to nearly 83%. The main reason is the interface migration of Li and I ions between the organic HTL and perovskite layer, which ultimately leads to the attenuation of performance.^37^

After that, perovskite film with a bandgap of 1.61 eV was sprayed onto the textured silicon solar cell. As can be seen from the SEM in Fig. S3,† the perovskite layer grows on the textured silicon surface and was compact and nonporous. In the cross-section SEM images, it can be found that perovskite grains are uniformly spread on the silicon textured surface. For both the top or the bottom of textured silicon, the perovskite layer was formed, and the morphology of the perovskite layer was perfectly repeated.

## Conclusions

In conclusion, the composite film of CsBr and PbI_2_ was evaporated by vacuum deposition, then an IPA mixed solution of FABr/FAI was sprayed ultrasonically to successfully prepare Cs_*y*_FA_1−*y*_PbI_*x*_Br_3−*x*_ perovskite film. The properties of the perovskite films were verified at different annealing temperatures by controlling the reaction conditions. The results show that the perovskite film with the best performance can be prepared under the annealing temperature of 170 °C, with good crystallinity and uniform morphology. In further research, we can easily adjust the Br/I ratio in the spray precursor solution to change the optical properties and appearance of the film. With the increase of FABr content in the solution, the colours of perovskite films gradually become lighter and the diffraction peaks in XRD pattern gradually move to a large angle. The optical bandgaps increase from 1.53 to 1.76 eV. Moreover, the requirement for the prepared substrate morphology is broader, which can realize the conformal growth of functional layers on large textured silicon cells.

## Author contributions

Conceived and designed the experiments: Shengquan Fu, Yong Peng. Performed the experiments: Shengquan Fu, Xinxin Yu. Analyzed the data: Shengquan Fu, Yueyue Xiao. Contributed reagents/materials/analysis tools: Tianxing Xiang, Fei Long, Junyan Xiao, Zhiliang Ku, Jie Zhong, Wei Li, Fuzhi Huang, Yibing Cheng.

## Conflicts of interest

There are no conflicts to declare.

## Supplementary Material

RA-011-D1RA02666F-s001

## References

[cit1] Yang P. X., Liu P., Ullah S., Wang J. M., Liu L. L., Yang S. E., Guo H. Z., Wang L. R., Chen Y. S. (2021). Sol. Energy.

[cit2] Yoon S. M., Min H., Kim J. B., Kim G., Lee K. S., Seok S. I. (2021). Joule.

[cit3] Chen B., Zheng X. P., Bai Y., Padture N. P., Huang J. S. (2017). Adv. Energy Mater..

[cit4] Futscher M. H., Ehrler B. (2016). ACS Energy Lett..

[cit5] Guo X. L., Ngai K. H., Qin M. C., Lu X. H., Xu J. B., Long M. Z. (2021). Nanotechnology.

[cit6] Li N. X., Luo Y. Q., Chen Z. H., Niu X. X., Zhang X., Lu J. Z., Kumar R., Jiang J. K., Liu H. F., Guo X., Lai B., Brocks G., Chen Q., Tao S. X., Fenning D. P., Zhou H. P. (2020). Joule.

[cit7] Valipour F., Yazdi E., Torabi N., Mirjalili B. F., Behjat A. (2020). J. Phys. D: Appl. Phys..

[cit8] Shao Z. P., Meng H. G., Du X. F., Sun X. H., Lv P. L., Gao C. Y., Rao Y., Chen C., Li Z. P., Wang X., Cui G. L., Pang S. P. (2020). Adv. Mater..

[cit9] Li N., Liu J. L., Li C., Li Y. J., Jia J. B., Wu Y. Q., Yu H. Q., Yuan B. L., Cao B. Q. (2020). ACS Sustainable Chem. Eng..

[cit10] Schutt K., Nayak P. K., Ramadan A. J., Wenger B., Lin Y. H., Snaith H. J. (2019). Adv. Funct. Mater..

[cit11] Chai G. D., Wang S. Z., Xia Z. G., Luo S. Q., Teng C., Yang T. B., Nie Z. X., Meng T. J., Zhou H. (2017). Semicond. Sci. Technol..

[cit12] Chou L. H., Wang X. F., Osaka I., Wu C. G., Liu C. L. (2018). ACS Appl. Mater. Interfaces.

[cit13] Lan D. H., Hong S. H., Chou L. H., Wang X. F., Liu C. L. (2018). J. Power Sources.

[cit14] Barrows A. T., Pearson A. J., Kwak C. K., Dunbar A. D. F., Buckley A. R., Lidzey D. G. (2014). Energy Environ. Sci..

[cit15] Heo J. H., Lee M. H., Jang M. H., Im S. H. (2016). J. Mater. Chem. A.

[cit16] Jiang Y., Remeika M., Hu Z. H., Juarez-Perez E. J., Qiu L. B., Liu Z. H., Kim T., Ono L. K., Son D. Y., Hawash Z., Leyden M. R., Wu Z. F., Meng L. Q., Hu J. S., Qi Y. B. (2019). Adv. Energy Mater..

[cit17] Yu X., Yan X., Xiao J., Ku Z., Zhong J., Li W., Huang F., Peng Y., Cheng Y. B. (2020). J. Chem. Phys..

[cit18] Hosseinian Ahangharnejhad R., Phillips A. B., Ghimire K., Koirala P., Song Z., Barudi H. M., Habte A., Sengupta M., Ellingson R. J., Yan Y., Collins R. W., Podraza N. J., Heben M. J. (2019). Sustainable Energy Fuels.

[cit19] Hossain M. I., Qarony W., Ma S., Zeng L., Knipp D., Tsang Y. H. (2019). Nano-Micro Lett..

[cit20] Barker A. J., Sadhanala A., Deschler F., Gandini M., Senanayak S. P., Pearce P. M., Mosconi E., Pearson A. J., Wu Y., Srimath Kandada A. R., Leijtens T., De Angelis F., Dutton S. E., Petrozza A., Friend R. H. (2017). ACS Energy Lett..

[cit21] Kim S. Y., Chae W. S., Na Y. J., Kim S. H., Lee S., Lee J. H., Heo Y. W. (2019). J. Alloys Compd..

[cit22] Jiang Y., Zhang H., Qiu X., Cao B. (2017). Mater. Lett..

[cit23] Liu J., Gao C., He X., Ye Q., Ouyang L., Zhuang D., Liao C., Mei J., Lau W. (2015). ACS Appl. Mater. Interfaces.

[cit24] Tsai C. H., Huang W. C., Wang W. S., Shih C. J., Chi W. F., Hu Y. C., Yu Y. H. (2017). J. Colloid Interface Sci..

[cit25] Chilvery A. K., Guggilla P., Batra A. K., Gaikwad D. D., Currie J. R. (2015). J. Photonics Energy.

[cit26] Liu D., Li Y., Yuan J., Hong Q., Shi G., Yuan D., Wei J., Huang C., Tang J., Fung M.-K. (2017). J. Mater. Chem. A.

[cit27] Zhang C., Lu Y.-N., Wu W.-Q., Wang L. (2021). Nano Energy.

[cit28] Li Z., Xiao C., Yang Y., Harvey S. P., Kim D. H., Christians J. A., Yang M., Schulz P., Nanayakkara S. U., Jiang C.-S., Luther J. M., Berry J. J., Beard M. C., Al-Jassim M. M., Zhu K. (2017). Energy Environ. Sci..

[cit29] Huang Z., Proppe A. H., Tan H., Saidaminov M. I., Tan F., Mei A., Tan C.-S., Wei M., Hou Y., Han H., Kelley S. O., Sargent E. H. (2019). ACS Energy Lett..

[cit30] Chen Q., Zhou H. P., Song T. B., Luo S., Hong Z. R., Duan H. S., Dou L. T., Liu Y. S., Yang Y. (2014). Nano Lett..

[cit31] Jacobsson T. J., Correa-Baena J. P., Anaraki E. H., Philippe B., Stranks S. D., Bouduban M. E. F., Tress W., Schenk K., Teuscher J., Moser J. E., Rensmo H., Hagfeldt A. (2016). J. Am. Chem. Soc..

[cit32] Yuan Y. B., Huang J. S. (2016). Acc. Chem. Res..

[cit33] Liu L., Huang S., Lu Y., Liu P. F., Zhao Y. Z., Shi C. B., Zhang S. Y., Wu J. F., Zhong H. Z., Sui M. L., Zhou H. P., Jin H. B., Li Y. J., Chen Q. (2018). Adv. Mater..

[cit34] Maiti A., Chatterjee S., Peedikakkandy L., Pal A. J. (2020). Sol. RRL.

[cit35] Tsai C. H., Huang W. C., Wang W. S., Shih C. J., Chi W. F., Hu Y. C., Yu Y. H. (2017). J. Colloid Interface Sci..

[cit36] Liu D. Y., Li Y., Yuan J. Y., Hong Q. M., Shi G. Z., Yuan D. X., Wei J., Huang C. C., Tang J. X., Fung M. K. (2017). J. Mater. Chem. A.

[cit37] Rivkin B., Fassl P., Sun Q., Taylor A. D., Chen Z., Vaynzof Y. (2018). ACS Omega.

