# Supplementary material for: Bandgap adjustment assisted preparation of >18% CsyFA1−yPbIxBr3−x-based perovskite solar cells using a hybrid spraying process
Source: RSC Adv. 2021 May 13;11(29):17595–602. doi: 10.1039/d1ra02666f (PMC9032767; doi:10.1039/d1ra02666f)
Supplement: RA-011-D1RA02666F-s001 [file RA-011-D1RA02666F-s001.pdf]

Electronic Supplementary Material (ESI) for RSC Advances.
This journal is © The Royal Society of Chemistry 2021

Supporting Information

Bandgap Adjustment Assisted Preparation >18% $Cs_yFA_{1-y}PbI_xBr_{3-x}$-based Perovskite Solar Cells in Hybrid Spraying Process

Shengquan Fu, [a] Yueyue Xiao, [a] Xinxin Yu, [a] Tianxing Xiang, [b] Fei Long, [c] Junyan Xiao, [a] Zhiliang Ku, [a] Jie Zhong, [a] Wei Li, [a] Fuzhi Huang, [a] Yong Peng*[a] and Yibing Cheng [a]

a. State Key Lab of Advanced Technologies for Materials Synthesis and Processing, Wuhan University of Technology, Wuhan 430070, P. R. China. E-mail: yongpeng@whut.edu.cn.

b. Wuhan University of Technology Advanced Engineering Technology Research Institute of Zhongshan City, Zhongshan 528400, P. R. China.

c. Guangxi Key Lab Opt & Elect Mat & Devices, Coll Mat Sci & Engn, Guilin University Technology, Guilin 541004, P. R. China.

The content of supporting information

1. Specific progress of perovskite solar cells prepared by spraying method.

2. Morphology of perovskite at different annealing temperatures.

3. Characteristics of perovskite solar cells.

4. The calculation formula of material properties.

5. Morphology of perovskite films deposited on textured silicon substrate.

1. Specific progress of perovskite solar cells prepared by spraying method

The process of perovskite solar cells prepared by ultrasonic spray method is summarized in **Table S1**.

Table S1. Specific progress of perovskite solar cells prepared by spraying method.

Method	Perovskite	*Eg* (eV)	PCE (%)	Author	Year
One-Step	$MAPbI_{3-x}Cl_x$	1.5-1.55	11.1%	A. T. Barrows	2014[1]
			13%	S. Das	2015[2]
			18.3%	J. H. Heo	2016[3]
	$MAPbI_3$	1.55	10.2%	M. Ramesh	2015[4]
			13.3%	S. C. Hong	2017[5]
			17.3%	S. Ulicna	2018[6]
	Triple Cation	~1.58	17.8%	J. E. Bishop	2018[7]
			18.5%	J. Su	2020[8]
			19.4%	J. E. Bishop	2020[9]
	Mixed Cation	1.45-1.5	15.9%	Y. Y. Jiang	2018[10]
			15.6%	Y. S. Chou	2019[11]
			17.6%	J. H. Heo	2019[12]
	$MAPbI_{3-x}Cl_x/MAPbI_{3-x}AC_x$	1.55	15.7%	J. G. Tait	2016[13]
Two-Step	$MAPbI_3$	1.55	11.7%	K. M. Boopathi	2015[14]
			12.5%	F. Li	2015[15]
			16%	H. B. Huang	2016[16]
	$FA_{1-x}Cs_xPbI_3$	1.5	14.9%	X. Xia	2016[17]
	$CsPbIBr_2$	2.05	6.3%	C. F. J. Lau	2016[18]
	Mixed Anion	1.55-1.6	13.4%	G. D. Chai	2017[19]
			16.2%	Y. Jiang	2019[20]
	$Cs_xFA_{1-x}PbI_yBr_{3-y}$	~1.58	18.2%	X. Yu	2020[21]

2. Morphology of perovskite at different annealing temperatures

As can be seen from the figure, when the annealing temperature is 110℃, except for a few large grains, a large number of small grains grow in the perovskite layer, the size of which is about 100-200nm. The surface of the film layer is obviously uneven. When the temperature reaches 130℃, the small grains grow up and the grain size becomes more uniform. This is mainly because the higher temperature accelerates the evaporation of the solution and contributes to the growth of perovskite grains.

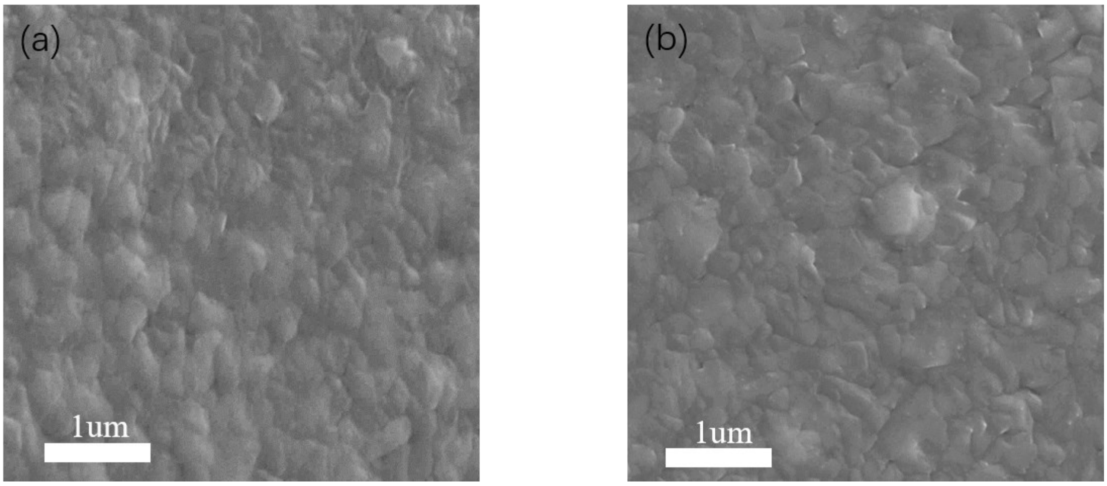

Fig. S1 SEM images(a, b) of surface morphology of perovskite annealed at 110℃ and 130℃.

3. Characteristics of perovskite solar cells

In **Fig. S2**(a), with the increase of Br content, the absorption curve of perovskite film gradually shifted to the left. It indicates that the peak of photon energy gradually increased, representing the gradual increase of film band gaps. **Fig. S2**(b) represents the dark current curve of perovskite with different bandgaps. It can be seen that except M = 0/0.2, the dark current of perovskite cells with other bandgaps basically has the same trend.

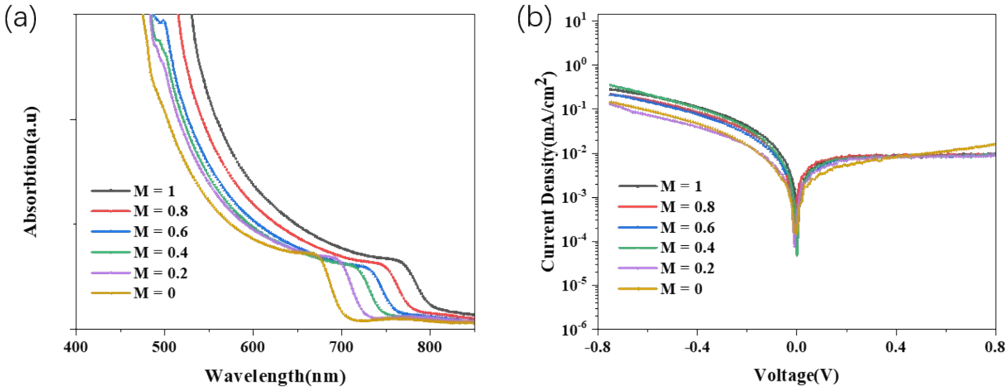

Fig. S2 The absorption curves (a), Semilog *J*–*V* characteristics (b) of the perovskite films at different Br content.

4. The calculation formula of material properties.

(1) The conversion formula of material absorbance and band gap.

For semiconductor materials, the relationship between the optical bandgap and the absorption coefficient is as follows **eqn.** (S1):

$$Ah\nu = C\,(h\nu - Eg)^{1/m} \qquad \text{(S1)}$$

Where hν is photon energy, C represents constant which depended on transition probability and m defines the type of transition. For direct bandgap semiconductors, such as perovskite, the m value is 1/2. Then, according to Beer-Lambert law[22], convert the previous formula to **eqn.** (S2)

$$(Ah\nu)^{1/2} = K\,(h\nu - Eg) \qquad \text{(S2)}$$

(2) The Bragg's law.

By Bragg's law[23], **eqn.** (S3) explain:

$$n\lambda = 2d\sin\theta \qquad \text{(S3)}$$

Where n represents the interference order, λ shows the wavelength of incident X-rays, d is the interplanar spacing and θ is the angle between the reflected or incident beam and the reflecting planes.

5. Morphology of perovskite films deposited on textured silicon substrate

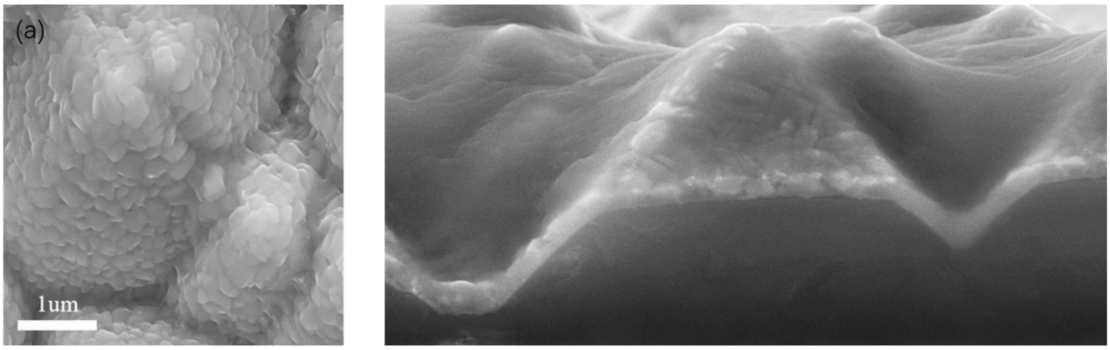

Fig. S3 SEM images of surface and cross-section of perovskite-textured silicon composite films.

Uncategorized References

1. A. T. Barrows, A. J. Pearson, C. K. Kwak, A. D. F. Dunbar, A. R. Buckley and D. G. Lidzey, *Energy Environ. Sci.*, 2014, **7**, 2944-2950.
2. S. Das, B. Yang, G. Gu, P. C. Joshi, I. N. Ivanov, C. M. Rouleau, T. Aytug, D. B. Geohegan and K. Xiao, *ACS Photonics*, 2015, **2**, 680-686.
3. J. H. Heo, M. H. Lee, M. H. Jang and S. H. Im, *Journal of Materials Chemistry A*, 2016, **4**, 17636-17642.
4. M. Ramesh, K. M. Boopathi, T. Y. Huang, Y. C. Huang, C. S. Tsao and C. W. Chu, *Acs Applied Materials & Interfaces*, 2015, **7**, 2359-2366.
5. S. C. Hong, G. Lee, K. Ha, J. Yoon, N. Ahn, W. Cho, M. Park and M. Choi, *Acs Applied Materials & Interfaces*, 2017, **9**, 7879-7884.
6. S. Ulicna, B. J. Dou, D. H. Kim, K. Zhu, J. M. Walls, J. W. Bowers and M. van Hest, *Acs Applied Energy Materials*, 2018, **1**, 1853-1857.
7. J. E. Bishop, J. A. Smith, C. Greenland, V. Kumar, N. Vaenas, O. S. Game, T. J. Routledge, M. Wong-Stringer, C. Rodenburg and D. G. Lidzey, *ACS Appl Mater Interfaces*, 2018, **10**, 39428-39434.
8. J. Su, H. K. Cai, J. T. Yang, X. F. Ye, R. Han, J. Ni, J. Li and J. J. Zhang, *Acs Applied Materials & Interfaces*, 2020, **12**, 3531-3538.
9. J. E. Bishop, C. D. Read, J. A. Smith, T. J. Routledge and D. G. Lidzey, *Sci Rep*, 2020, **10**, 6610.
10. Y. Y. Jiang, C. C. Wu, L. R. Li, K. Wang, Z. Tao, F. Gao, W. F. Cheng, J. T. Cheng, X. Y. Zhao, S. Priya and W. W. Deng, *Nano Energy*, 2018, **53**, 440-448.
11. Y. S. Chou, L. H. Chou, A. Z. Guo, X. F. Wang, I. Osaka, C. G. Wu and C. L. Liu, *Acs Sustainable Chemistry & Engineering*, 2019, **7**, 14217-14224.
12. J. H. Heo, K. Im, J. Kim and S. H. Im, *ACS Appl Mater Interfaces*, 2020, **12**, 886-892.
13. J. G. Tait, S. Manghooli, W. Qiu, L. Rakocevic, L. Kootstra, M. Jaysankar, C. A. M. de la Huerta, U. W. Paetzold, R. Gehlhaar, D. Cheyns, P. Heremans and J. Poortmans, *Journal of Materials Chemistry A*, 2016, **4**, 3792-3797.
14. K. M. Boopathi, M. Ramesh, P. Perumal, Y. C. Huang, C. S. Tsao, Y. F. Chen, C. H. Lee and C. W. Chu, *Journal of Materials Chemistry A*, 2015, **3**, 9257-9263.
15. F. Li, C. Bao, H. Gao, W. Zhu, T. Yu, J. Yang, G. Fu, X. Zhou and Z. Zou, *Materials Letters*, 2015, **157**, 38-41.
16. H. B. Huang, J. J. Shi, L. F. Zhu, D. M. Li, Y. H. Luo and Q. B. Meng, *Nano Energy*, 2016, **27**, 352-358.
17. X. Xia, W. Y. Wu, H. C. Li, B. Zheng, Y. B. Xue, J. Xu, D. W. Zhang, C. X. Gao and X. Z. Liu, *Rsc Advances*, 2016, **6**, 14792-14798.
18. C. F. J. Lau, X. F. Deng, Q. S. Ma, J. H. Zheng, J. S. Yun, M. A. Green, S. J. Huang and A. W. Y. Ho-Baillie, *Acs Energy Letters*, 2016, **1**, 573-577.
19. G. D. Chai, S. Q. Luo, H. Zhou and W. A. Daoud, *Materials & Design*, 2017, **125**, 222-229.
20. Y. Jiang, M. Remeika, Z. H. Hu, E. J. Juarez-Perez, L. B. Qiu, Z. H. Liu, T. Kim, L. K. Ono, D. Y. Son, Z. Hawash, M. R. Leyden, Z. F. Wu, L. Q. Meng, J. S. Hu and Y. B. Qi, *Advanced Energy Materials*, 2019, **9**, 1803047.
21. X. Yu, X. Yan, J. Xiao, Z. Ku, J. Zhong, W. Li, F. Huang, Y. Peng and Y. B. Cheng, *J Chem Phys*, 2020, **153**, 014706.

22. G. Strangman, M. A. Franceschini and D. A. Boas, *Neuroimage*, 2003, **18**, 865-879.
23. H. N. Chapman and S. Bajt, *J. Opt.*, 2020, **22**, 115610.
